# Epicardial Potential in the Left Atrium During Posterior Wall Isolation in Persistent Atrial Fibrillation

**DOI:** 10.19102/icrm.2023.14074

**Published:** 2023-07-15

**Authors:** Ankita Dubey, Ammar Ahmed, Harshil Patel, Jessica Wrobel, Julia Donlon, Harini Lakshman, Dipak Shah

**Affiliations:** ^1^Department of Internal Medicine, Ascension Providence Hospital, Michigan State University/College of Human Medicine, Grand Rapids, MI, USA; ^2^Department of Cardiovascular Disease, Ascension Providence Hospital, Michigan State University/College of Human Medicine, Southfield, MI, USA; ^3^Abbott Laboratories, Abbott Park, IL, USA

**Keywords:** Atrial fibrillation, high-density mapping, posterior wall isolation, pulmonary vein isolation

## Abstract

Pulmonary vein isolation (PVI) is used for rhythm control in atrial fibrillation (AF). Posterior wall isolation (PWI) is often an adjunct to PVI. Successful PWI is limited by esophageal location, epicardial bridging signals, tissue thickness, and mapping catheter resolution. High-density grid mapping catheters can assist with PWI. Here, we report a case of a 71-year-old woman with persistent AF who underwent PVI and PWI with high-density grid mapping catheters, thus demonstrating the use of omnipolar technology in facilitating successful PWI.

## Introduction

Catheter ablation for atrial fibrillation (AF) is an effective therapy to achieve rhythm control.^1–3^ Systematic review and meta-analysis have shown that catheter ablation as a first-line therapy for paroxysmal AF results in a 38% reduction in recurrent atrial arrhythmias.^4,5^ The cornerstone of AF ablation is antral pulmonary vein (PV) isolation (PVI); however, the delineation of the antral PV from the posterior wall (PW) is often difficult. In addition, the PW embryologically originates from the same tissue as the PV. There are limited data to support the idea that patients with persistent AF who undergo adjunctive PW isolation (PWI) have improved maintenance of sinus rhythm compared to patients undergoing PVI alone.^6–11^ Multielectrode high-density (HD) catheters have demonstrated that effective PVI alone can be challenging.^12^ The same appears to be true for PWI as the success of a simple box lesion set is limited by tissue thickness and epicardial connections.^13^ We present the case of a patient undergoing AF ablation with both PVI and PWI with a likely epicardial connection delineated by an HD grid catheter using omnipolar mapping technology (OT).

## Case presentation

**Figure 1:**
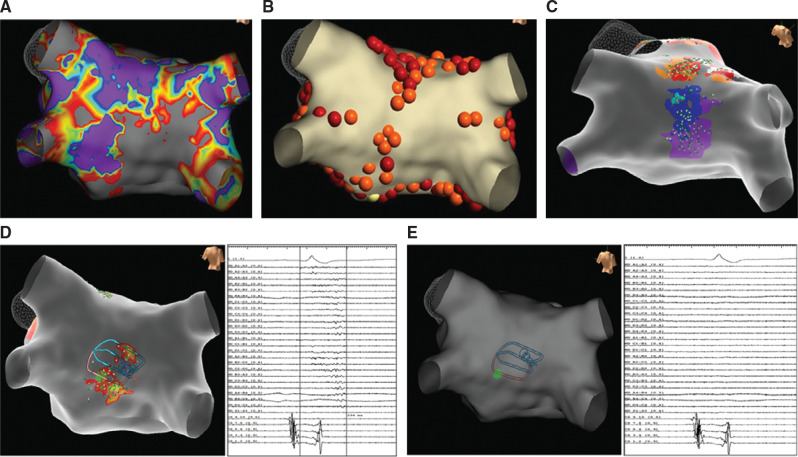
**A:** Voltage map prior to ablation displaying significant scarring. **B:** Circumferential pulmonary vein isolation and pulmonary wall box lesion set. **C:** Activation map using omnipolar mapping technology displaying the roof and floor block with a focal pulmonary wall source. **D:** Fractionated, low-amplitude high-density grid signal on the pulmonary wall spanning 235 ms. **E:** Electograms on a high-density grid and voltage map revealing pulmonary wall isolation after ablation at fractionated signal.

A 71-year-old woman with symptomatic persistent AF underwent AF ablation with the Abbott EnSite X mapping system with the Sensor Enabled™ Advisor™ HD Grid mapping catheter and TactiCath™ SE contact force ablation catheter (Abbott, Chicago, IL, USA). Given her persistent AF as well as the scarring seen around her PVs and PW **(Figure 1A)**, PWI in addition to PVI was performed with a box lesion set **(Figure 1B)**. Although OT and a color-coded isochronal activation map revealed PVI and block across the floor and roof of our box lesion set, the PW was not isolated. The block across the roof is demonstrated by the non-contiguous color scheme on the isochronal activation map as well as vectors splitting on the roof in two directions **(Figure 1C)**. Separately, the floor block is demonstrated by the absence of vectors or voltage on the floor **(Figure 1C)**. **Figure 1D** shows fractionated signals on the PW bounded by the roof and floor lines. The earliest signal seen on the HD Grid map revealed by the green lesion is in the midpoint of the fractionated voltage island. This “vector chaos” suggests an epicardial source **(Figure 1D)**. A single ablation lesion at this fractionated signal isolated the PW **(Figure 1E)**.

## Discussion

With 46.3 million patients currently diagnosed with AF worldwide and an expected increase in disease burden forthcoming, AF represents a growing health concern.^14^ The multicenter Early Treatment of Atrial Fibrillation for Stroke Prevention Trial (EAST-AFNET 4) demonstrated that rhythm-control strategies with either anti-arrhythmic drug or ablation are superior to the standard rate-control regimen.^15^ However, catheter ablation of persistent AF remains an ongoing challenge due to a high recurrence rate with an estimated 45% of patients maintaining sinus rhythm over 5 years after multiple ablation procedures.^16^ The pathophysiology of AF, which also involves endomysial fibrosis within the epicardial layer, causes electrical dissociation between the endocardial and epicardial networks. Due to this dissociation, there is an increased risk of developing long-standing AF, supporting the need for early catheter ablation strategies (PVI, PVI + PWI).^17^

The PW of the left atrium is of particular concern for the initiation and maintenance of AF.^18^ It shares similar embryological origins with the PVs, and, with significant heterogeneity in the orientation of myocardial fibers, it is likely a significant arrhythmogenic source. Therefore, additional substrate ablation has been considered as a viable method to reduce recurrence.^18^ The 2015 Substrate and Trigger Ablation for Reduction of Atrial Fibrillation Trial Part II (STAR AF II) studied PVI alone versus PVI with the addition of either fractionated electrogram ablation or linear ablation across the roof of the left atrium and the mitral valve isthmus. This study found no reduction in AF recurrence using either technique in addition to PVI. However, neither technique isolated the PW.^19^ Though multiple studies have assessed the benefit of adjunctive PWI, the outcomes have varied.^20–22^ A large secondary analysis involving 26 clinical studies (>3000 patients) showed that patients with persistent AF who undergo adjunctive PWI have a lower risk of recurrence of AF and all atrial arrhythmias.^22^ Recently, the Catheter Ablation for Persistent Atrial Fibrillation: A Multicenter Randomized Trial of Pulmonary Vein Isolation Versus PVI with Posterior Left Atrial Wall Isolation (CAPLA) study compared patients with symptomatic persistent AF undergoing PVI alone versus PVI + PWI using radiofrequency ablation. The study revealed no significant benefit in atrial arrhythmia recurrence at 12 months of follow-up.^23^ While theoretically viable, proving the benefit of PWI in randomized trials has been challenging.^23^ CAPLA only followed up on patients for 12 months. Additionally, PWI is technically challenging and may hold greater benefit in patients with anatomy predisposed to PW arrhythmogenicity. Moreover, as demonstrated by our case, what appears to be an apparent isolation of the PW using conventional catheters may have undetected residual low-voltage potentials requiring an HD catheter for identification and subsequent ablation.

Achieving complete isolation of the PW is more challenging than previously believed. With the advent of HD catheters, we have learned that remnant low-voltage activity can be appreciated after PVI.^24,25^ It is still unclear whether ablation of the activity revealed by the HD catheters will result in prolonged freedom from AF compared to in those patients who underwent the procedure with standard non-HD catheters instead.^25^ In our case, we were able to complete PWI after ablation of the likely epicardial signal. On follow-up, the patient continues to be in sinus rhythm.

## Conclusion

Our case emphasizes that, despite the roof and floor block of the PW, a residual low-voltage epicardial connection can be identified using the HD Grid catheter with OT. What was thought previously to be complete PVI and PWI with non-HD catheters is likely overestimated. Whether a more complete PVI plus PWI procedure with HD catheters translates to reduced recurrence rates of AF is yet to be seen and needs to be investigated in future studies.

## References

[1] January CT, Wann LS, Calkins H (2019). 2019 AHA/ACC/HRS focused update of the 2014 AHA/ACC/HRS guideline for the management of patients with atrial fibrillation: a report of the American College of Cardiology/American Heart Association Task Force on Clinical Practice Guidelines and the Heart Rhythm Society. J Am Coll Cardiol.

[2] Chen S, Pürerfellner H, Meyer C (2020). Rhythm control for patients with atrial fibrillation complicated with heart failure in the contemporary era of catheter ablation: a stratified pooled analysis of randomized data. Eur Heart J.

[3] Hindricks G, Potpara T, Dagres N (2021). 2020 ESC guidelines for the diagnosis and management of atrial fibrillation developed in collaboration with the European Association for Cardio-Thoracic Surgery (EACTS): the Task Force for the diagnosis and management of atrial fibrillation of the European Society of Cardiology (ESC) Developed with the special contribution of the European Heart Rhythm Association (EHRA) of the ESC. Eur Heart J.

[4] Turagam MK, Musikantow D, Whang W (2021). Assessment of catheter ablation or antiarrhythmic drugs for first-line therapy of atrial fibrillation: a meta-analysis of randomized clinical trials. JAMA Cardiol.

[5] Hakalahti A, Biancari F, Nielsen JC, Raatikainen MJ (2015). Radiofrequency ablation vs. antiarrhythmic drug therapy as first line treatment of symptomatic atrial fibrillation: systematic review and meta-analysis. Europace.

[6] Kim JS, Shin SY, Na JO (2015). Does isolation of the left atrial posterior wall improve clinical outcomes after radiofrequency catheter ablation for persistent atrial fibrillation?: a prospective randomized clinical trial. Int J Cardiol.

[7] Lee JM, Shim J, Park J (2019). The electrical isolation of the left atrial posterior wall in catheter ablation of persistent atrial fibrillation. JACC Clin Electrophysiol.

[8] Yamaji H, Higashiya S, Murakami T (2020). Efficacy of an adjunctive electrophysiological test-guided left atrial posterior wall isolation in persistent atrial fibrillation without a left atrial low-voltage area. Circ Arrhythm Electrophysiol.

[9] Aryana A, Allen SL, Pujara DK (2021). Concomitant pulmonary vein and posterior wall isolation using cryoballoon with adjunct radiofrequency in persistent atrial fibrillation. JACC Clin Electrophysiol.

[10] Pak HN, Park J, Park JW (2020). Electrical posterior box isolation in persistent atrial fibrillation changed to paroxysmal atrial fibrillation: a multi-center, prospective, randomized study. Circ Arrhythm Electrophysiol.

[11] Salih M, Darrat Y, Ibrahim AM (2020). Clinical outcomes of adjunctive posterior wall isolation in persistent atrial fibrillation: a meta analysis. J Cardiovasc Electrophysiol.

[12] Segerson NM, Lynch B, Mozes J (2018). High-density mapping and ablation of concealed low-voltage activity within pulmonary vein antra results in improved freedom from atrial fibrillation compared to pulmonary vein isolation alone. Heart Rhythm.

[13] Hocini M, Jaïs P, Sanders P (2005). Techniques, evaluation, and consequences of linear block at the left atrial roof in paroxysmal atrial fibrillation: a prospective randomized study. Circulation.

[14] Kornej J, Borschel CS, Benjamin EJ, Schnabel RB (2020). Epidemiology of atrial fibrillation in the 21st century: novel methods and new insights. Circ Res.

[15] Kirchhof P, Camm A, Goette A (2020). Early rhythm-control therapy in patients with atrial fibrillation. N Engl J Med.

[16] Tilz RR, Rillig A, Thum A-M (2012). Catheter ablation of long-standing persistent atrial fibrillation: 5-year outcomes of the Hamburg Sequential Ablation Strategy. J Am Coll Cardiol.

[17] Verheule S, Eckstein J, Linz D (2014). Role of endo-epicardial dissociation of electrical activity and transmural conduction in the development of persistent atrial fibrillation. Prog Biophys Mol Biol.

[18] Tahir KS, Mounsey JP, Hummel JP (2018). Posterior wall isolation in atrial fibrillation ablation. J Innov Card Rhythm Manag.

[19] Verma A, Jiang CY, Betts TR (2015). Approaches to catheter ablation for persistent atrial fibrillation. N Engl J Med.

[20] Thiyagarajah A, Kadhim K, Lau DH (2019). Feasibility, safety, and efficacy of posterior wall isolation during atrial fibrillation ablation: a systematic review and meta-analysis. Circ Arrhythm Electrophysiol.

[21] Lupercio F, Lin AY, Aldaas OM (2020). Role of adjunctive posterior wall isolation in patients undergoing atrial fibrillation ablation: a systematic review and metaanalysis. J Interv Cardiac Electrophysiol.

[22] Jiang X, Liao J, Ling Z (2022). Adjunctive left atrial posterior wall isolation in treating atrial fibrillation: insight from a large secondary analysis. JACC Clin Electrophysiol.

[23] Chieng D, Sugumar H, Ling LH (2022). Catheter ablation for persistent atrial fibrillation: a multicenter randomized trial of pulmonary vein isolation (PVI) versus PVI with posterior left atrial wall isolation (PWI) – the CAPLA study. Am Heart J.

[24] Porterfield C, Gora PJ, Wystrach A (2020). Confirmation of pulmonary vein isolation with high-density mapping: comparison to traditional workflows. J Atr Fibrillation.

[25] Huo Y, Gaspar T, Schönbauer R (2022). Low-voltage myocardium-guided ablation trial of persistent atrial fibrillation. NEJM Evidence.

